# Identification of Leaf-Scale Wheat Powdery Mildew (*Blumeria graminis* f. sp. *Tritici*) Combining Hyperspectral Imaging and an SVM Classifier

**DOI:** 10.3390/plants9080936

**Published:** 2020-07-24

**Authors:** Jinling Zhao, Yan Fang, Guomin Chu, Hao Yan, Lei Hu, Linsheng Huang

**Affiliations:** 1 National Engineering Research Center for Agro-Ecological Big Data Analysis & Application, Anhui University, Hefei 230601, China; 2 School of Electronics and Information Engineering, Anhui University, Hefei 230601, China; P18301127@stu.ahu.edu.cn (Y.F.); P19301110@stu.ahu.edu.cn (G.C.); P19201087@stu.ahu.edu.cn (H.Y.); P19301115@stu.ahu.edu.cn (L.H.)

**Keywords:** disease severity, hyperspectral imaging, probabilistic neural network, successive projections algorithm, support vector machine, wheat powdery mildew

## Abstract

Powdery mildew (PM, *Blumeria graminis* f. sp. *tritici*) is a devastating disease for wheat growth and production. It is highly meaningful that the disease severities can be objectively and accurately identified by image visualization technology. In this study, an integral method was proposed based on a hyperspectral imaging dataset and machine learning algorithms. The disease severities of wheat leaves infected with PM were quantitatively identified based on hyperspectral images and image segmentation techniques. A technical procedure was proposed to perform the identification and evaluation of leaf-scale wheat PM, specifically including three primary steps of the acquisition and preprocessing of hyperspectral images, the selection of characteristic bands, and model construction. Firstly, three-dimensional reduction algorithms, namely principal component analysis (PCA), random forest (RF), and the successive projections algorithm (SPA), were comparatively used to select the bands that were most sensitive to PM. Then, three diagnosis models were constructed by a support vector machine (SVM), RF, and a probabilistic neural network (PNN). Finally, the best model was selected by comparing the overall accuracies. The results show that the SVM model constructed by PCA dimensionality reduction had the best result, and the classification accuracy reached 93.33% by a cross-validation method. There was an obvious improvement of the identification accuracy with the model, which achieved an 88.00% accuracy derived from the original hyperspectral images. This study can provide a reference for accurately estimating the disease severity of leaf-scale wheat PM and other plant diseases by non-contact measurement technology.

## 1. Introduction

As one of the main cereal crops, wheat has been widely grown in northern China. In recent years, various wheat diseases, such as powdery mildew (PM, *Blumeria graminis* f. sp. *tritici*), stripe rust (*Puccinia striiformis* f. sp. *tritici*), and wheat scab (*Fusarium graminearum* Schwabe), have occurred due to various pathogens and the weather in this region favors the occurrence and spreading of such diseases [1]. The wheat grain yield and quality have been greatly affected, threatening food security. It is becoming increasingly important to assess and control the disease epidemic. When wheat PM occurs, it is important to derive the disease severity from the symptoms, which can provide an essential reference for population virulence and cultivar resistance. Traditionally, more time and labor have been required for phytopathologists to estimate the infection. It is inevitable that a comparison of multiple experiments is difficult and some mistakes may be made by humans. With the development of remote sensing and image processing techniques, the quantitative accuracy has been greatly improved [2,3]. Ma et al. [4] jointly used the AdaBoost model and a max-relevance and min-redundancy (mRMR) algorithm to detect wheat PM with an overall accuracy of 88.4%. Steddom et al. [5] used a multi-band spectrometer to accurately assess the incidence of brown spot disease in beet, improving the accuracy of brown spot surveillance. Adams et al. [6] monitored soybean chlorosis by studying the spectra of soybean leaves.

After wheat leaves are infected by PM, some symptoms can be visually observed when the severity is relatively serious. Nevertheless, it is usually too late to prevent and control the disease. In fact, some of the infection symptoms can be detected in the near-infrared (NIR) wavelength region rather than the visible range [7]. The PM pathogen can cause changes in the leaf pigment content, cell structure, and leaf water, and the spectral responses differ in different spectral domains accordingly. When the wheat leaves are infected by PM, in comparison with the visible range, the spectral differences are sharper in the NIR wavelength region. Several ground-based remote sensing systems and spectral indices have been developed for plant disease diagnosis and detection [8,9,10,11,12]. For example, Zhang et al. detected the leaf-scale PM of winter wheat using leaf level hyperspectral measurements derived from an Analytical Spectral Device (ASD, Inc., Boulder, CO, USA) spectroradiometer [13]. Shi et al. adopted spectral indices and kernel discriminant analysis to detect and discriminate the pests and diseases in winter wheat using an ASD FieldSpec spectrometer [14]. Non-imaging hyperspectral data are usually used in studies of crop disease monitoring and diagnosis.

More attention has been paid to hyperspectral imaging technology due to its integration of spectra and images. In comparison with visible-band and wide-band images, hyperspectral imaging technology can provide more objective and accurate results due to its integration of images and spectra [15]. It has been widely used in the detection of crop diseases in recent years. Williams et al. [16] successfully detected the fusarium disease on corn by using hyperspectral imaging technology. Bauriege et al. [17] used hyperspectral imaging technology to detect early wheat *Fusarium* diseases, and the accuracy of disease level detection was 87%. Del Fiore et al. [18] conducted early detection research on corn fungal diseases using hyperspectral imaging technology. Liu et al. [19] established a model for investigating the relationship between the severity of rice blight and the spectrum based on a spectral study of rice ears. Graeff et al. [20] analyzed the spectra of wheat leaves and found that powdery mildew had a strong spectral response in some sensitive bands. Therefore, hyperspectral imaging can provide a fine comparison of healthy and diseased wheat plants.

After being infected by PM, pustules with a light white (sometimes light yellow) color can be observed on wheat leaves [21]. The significant spectral differences between healthy leaves and infected ones can be accurately identified by selecting pure regions of interest (ROIs) on high-resolution hyperspectral images. Comprehensive information combining the visible and NIR wavelengths can provide a more rational and accurate diagnosis of the disease. In addition, a leaf-scale diagnosis study is an appropriate way of investigating the disease features compared with canopy and regional scales. Therefore, the objectives of this study were (1) to examine the effect on identifying characteristic bands before and after spectral smoothing during the acquisition of hyperspectral data, (2) to compare the three-dimensional reduction methods for selecting spectral bands sensitive to wheat PM, and (3) to identify the severity levels of PM on wheat leaves by comparing the classification accuracies of three inversion models.

## 2. Materials and Methods

### 2.1. Technical Procedure

**Figure 1:**
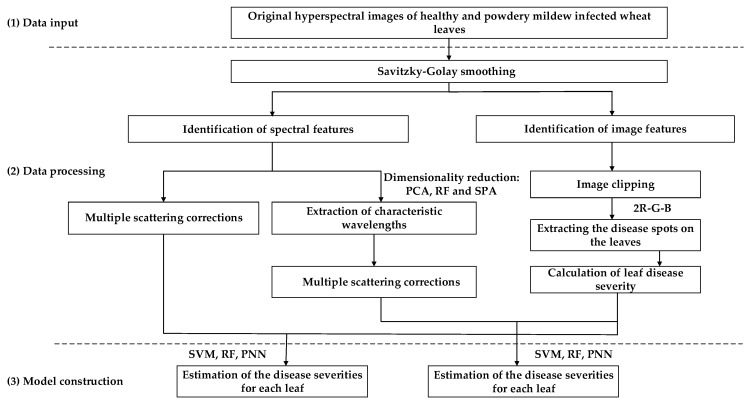
The overall technical workflow of the study.

Three primary steps were required to carry out the technical procedure (Figure 1). The original hyperspectral images of healthy and diseased wheat leaves were first smoothed by the Savitzky–Golay (S-G) filter, and the spectral and image features were then identified. To reduce the calculation time and highly-significant correlation among neighboring bands, three-dimensional reduction methods were comparatively used, namely principal component analysis (PCA), random forest (RF), and the successive projections algorithm (SPA). The average spectra of original and dimensionally-reduced wheat leaves were extracted by multiple scattering corrections (MSC). After removing the regions of thumb tacks, the disease spots on the leaves were extracted in the 2R-G-B color difference RGB color space. The disease severity for each leaf was calculated by the ratio between disease spots and the entire leaf. Considering the spectral and corresponding disease severity simultaneously, three detection models were comparatively constructed by the support vector machine (SVM), RF, and the probabilistic neural network (PNN).

### 2.2. Acquision and Preprocessing of Hyperspectral Images

#### 2.2.1. Experimental Design

The experiment was carried out at the experimental field of Beijing Academy of Agriculture and Forestry Sciences (39.93° N, 116.27° E), China [22]. To ensure disease incidence, the wheat variety Jingdong-12 susceptible to PM was selected and grown under normal water and fertilizer management. The symptoms could be gradually observed with the increase of disease severities and the development of growth stages. To improve the contrast enhancement between healthy and diseased wheat leaves, data collection was performed during the grain-filling stage, which is an essential period that affects the yield and quality of wheat. Wheat plants were first assessed by an experienced pathologist in the field, without destroying them. A total of 75 sample leaves, including 60 diseased leaves and 15 healthy leaves, were picked. To keep the wheat leaves fresh, a portable fridge was used to store them. In addition, the hyperspectral imaging device was installed in a dark room near the experimental site and fixed over a sampling platform covered with a black cloth. Leaves with different severities were fixed on the cloth by thumb tacks.

#### 2.2.2. Acquisition of Hyperspectral Image Cubes

**Figure 2:**
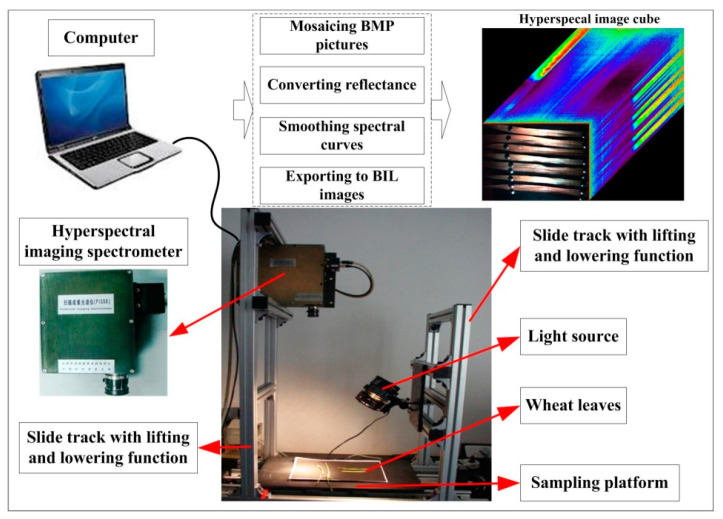
The devices used to measure the hyperspectral reflectance of wheat leaves.

Three necessary procedures had to be performed to acquire and process the hyperspectral images, including mosaicing, reflectance conversion, and spectral smoothing (Figure 2) [23]. A ground-based pushbroom imaging spectrometer (PIS) was used to collect the hyperspectral images. PIS acquires images by linear array pushbroom imaging technology. It was jointly developed by the Beijing Research Center for Information Technology in Agriculture and the University of Science and Technology of China. The sensor can collect a hyperspectral image (cube) and pixel-by-pixel spectral information within the effective wavelengths of 400–1000 nm, with a spectral resolution of 2 nm and a sampling interval of 0.7 nm. It has a field of view of 16° and a spatial resolution of 5–10 mm. A hyperspectral image of 1400 (spatial dimension) × 1024 (spectral dimension) can be acquired for each scan. There are various illumination intensities in different wavelengths, due to the existence of dark current. Some noises can be caused in the low-illumination spectral bands. To reduce the noises and perform reflectance conversion, the standard reference panel must be used to optimize the instrument before and after collecting the spectra.

#### 2.2.3. Data Preprocessing

**Figure 3:**
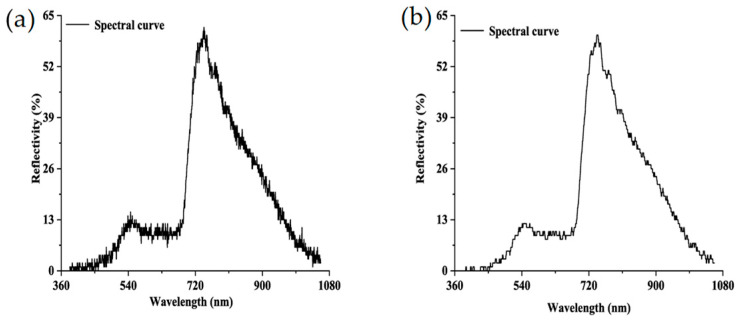
Comparison of hyperspectral reflectance curves between (**a**) original and (**b**) Savitzky–Golay (S-G) filter-smoothed data.

When the Bitmap (BMP) format pictures were mosaiced to generate an entire image of a group of wheat leaves, reflectance conversion was conducted using Equation (1). Last but not least, to reduce the random noises during data collection, the S-G filter was used to improve the spectral smoothness in the Environment for Visualizing Images (ENVI). Firstly, the spectral mean values of 75 hyperspectral leaves were extracted by calculating all of the pixel values. The derivative function was used to correct the baseline effects, which could amplify and resolve the overlapped signal. In SG smoothing, the window size and polynomial order must be specified. The window size must be an odd number and was set to 21 here, and the polynomial order must be less than the window length and was set to 2 in our experiment [24]. A comparison of the spectral curves before and after the S-G smoothing filter is shown in Figure 3.

(1) ρ=a×DN+b,

where *ρ* is the spectral reflectance, a and b are the coefficients, and DN is the digital number for a pixel in the original image. The values of a and b can be ensured by the least-squares method when incorporating the measured spectral value and corresponding DN into Equation (1). The *ρ* of the hyperspectral image can be calculated accordingly.

#### 2.2.4. Determination of Disease Severity

**Figure 4:**
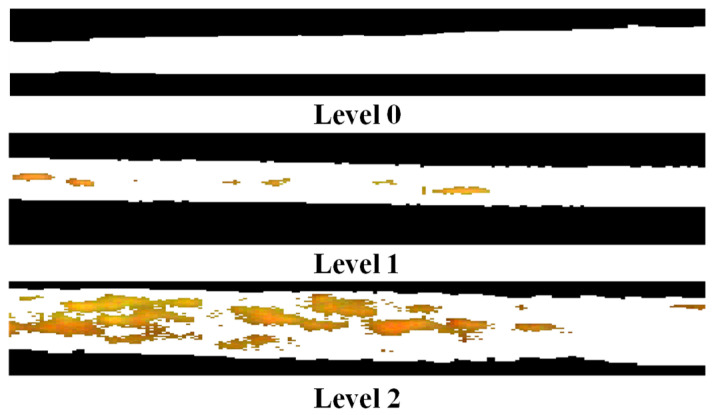
The segmentation results of disease spots for the three levels.

According to the rules for the investigation and forecasting of wheat PM of China (NY/T 613–2002), the disease severity (DS) can be divided into eight levels based on the ratio of diseased spots to total leaf area (Equation (2)), i.e., 1%, 5%, 10%, 20%, 40%, 60%, 80%, and 100%. Nevertheless, the spectral differences are not significant enough for some levels, especially for neighboring levels. In our study, to enhance the spectral comparison of different levels, the eight levels were recategorized into three levels: Healthy (Level 0, DS < 5%), slight (Level 1, 5% < DS < 40%,), and serious (Level 2, DS > 40%). The disease spots were identified using the threshold segmentation method and are shown in the 2R-G-B color space (Figure 4). It is obvious that the model can identify the disease spots well.

(2) $$D=\frac{A_{D}}{A_{T}}\times 100\%$$

Here, *D* is the disease severity of the wheat leaf, *A*_D_ is the area of PM disease spots on the leaf, and *A*_T_ is the total area of the leaf.

### 2.3. Methods for Hyperspectral Dimensionality Reduction

The original hyperspectral image ranges from 400 to 1000 nm and is divided into 1024 bands. When all of the bands are used as the input variables, a long computation time and advanced computer configuration are required. In addition, in such a high-dimensional space, it is inevitable that there is a strong correlation among multiple bands. The information redundancy and random noises will affect the model sensitivity and reduce the identification accuracy [25,26]. In general, there is no universal dimensionality reduction method for any application scene. Consequently, it is highly necessary to select the wavelength bands that are sensitive to PM. In this study, three methods were comparatively used, namely principal component analysis (PCA), random forest (RF), and the successive projections algorithm (SPA).

#### 2.3.1. PCA

PCA is a method that removes redundant information between bands and compresses multi-band image information to a few bands that are more effective than the original bands. It is a statistical method widely used in unsupervised dimensionality reduction [27]. Linear transformation is used to transform the original data into a set of linearly independent representations for each dimension, which can be used to extract the main feature components of the objective data. In essence, the direction with the largest variance is used as the main feature to make sure that there are no correlations in different orthogonal directions [28,29]. Fewer new variables are selected to replace the original variables under the premise of keeping most of the spectral information. In this study, the first three principal components contained 99.21% of the variance information, so they were retained. The peaks and valleys were selected as the characteristic wavelength bands.

#### 2.3.2. SPA

The successive projections algorithm (SPA) is an effective variable-selection technique that has attracted increasing interest in hyperspectral remote sensing [30,31,32]. The primary purpose is to select wavelengths in which the information content is minimally redundant to solve collinearity problems. It is a forward selection method starting with one wavelength, and then incorporates a new one at each iteration, until a specified number *N* of wavelengths is reached [33]. The specific steps of the SPA are shown in Table 1.

### 2.4. Modeling Methods

The seventy-five samples were divided into five groups. A group was selected as the test set and the remaining four groups were used as the training set. Three detection models using the three-dimensional reduction methods were constructed and compared. Cross-validation was used to evaluate the accuracy of the constructed models. The ROI tool in ENVI was used to create ROIs to verify the accuracy of disease spot segmentation.

#### 2.4.1. SVM

The SVM is a machine learning method based on the statistical learning theory [34,35]. It can maximize the separation or margin between samples of different classes by constructing a set of hyperplanes. There are several unique advantages in addressing issues such as small samples, non-linearity, and high-dimensional pattern recognition problems. The phenomena of “dimensional disaster” and “over-learning” can be avoided to a great degree. Several parameters should be evaluated and specified, including the kernel function, values for gamma, and cost. In this study, the radial basis function (RBF) (Equation (3)) was used as the kernel function. The cross-validation was applied to identify the best parameters.

(3) $$K\left(x_{i},x\right)=\exp\left(-\frac{\left\|x-x_{i}\right\|}{2\sigma^{2}}\right)$$

Here, *x_i_* is the input training data and *σ* is the kernel parameter.

#### 2.4.2. RF

RF, consisting of multiple decision trees, is a classification or regression method. It was proposed by Leo Breiman in 2001 [36]. The decision trees are created on a bootstrap sample of training data by using a random selection of variable subsets. The *n* training sets can be obtained after n-time sampling. The *n* decision tree models are constructed based on each new training set and a random forest can be generated. Every tree of the forest then votes to determine the sample’s class, and a majority vote makes the final decision [37,38]. In our study, the RF classifier was built with the recommended values by Breiman, with the number of decision trees being 500 and default values for other parameters.

#### 2.4.3. PNN

A probabilistic neural network (PNN) is a direct continuation of the work on Bayes classifiers [39]. It is a branch of radial basis function networks that belongs to a feedforward neural network. There are four layers, including input units, pattern units, summation units, and output units. The nodes are allocated in the three layers after the inputs. There is one pattern node for each training example. Each summation node receives the outputs from pattern nodes associated with a given class. The output nodes are binary neurons that produce the classification decision. More precisely, the PNN is interpreted as a function that approximates the probability density of the underlying examples’ distribution, rather than the examples directly by fitting [40]. A Gaussian function (Equation (4)) is used to connect the input layer and pattern layer. The matching degree is calculated between neurons of the two layers. Then, the specific class of input samples can be ensured by cumulatively adding and averaging the matching degree for each category.

(4) $$y_{g}\left(x;\sigma \right)=\frac{1}{l_{g}{\left(2\pi \right)}^{n/2}\sigma^{n}}\sum_{i=1}^{l_{g}}\exp\left(-\sum_{j=1}^{n}\frac{{\left({x_{ij}}^{\left(g\right)}-x_{j}\right)}^{2}}{2\sigma^{2}}\right)$$

Here, *l_g_* is the number of category *g*; *n* is the feature number; *σ*, the unique adjustable factor, is the smoothing parameter located between 0 and 1; and *x_ij_* is the *j*th datum of the *i*th neuro for *g*.

## 3. Results and Discussion

### 3.1. Characterizaion of the Disease Severities

**Figure 5:**
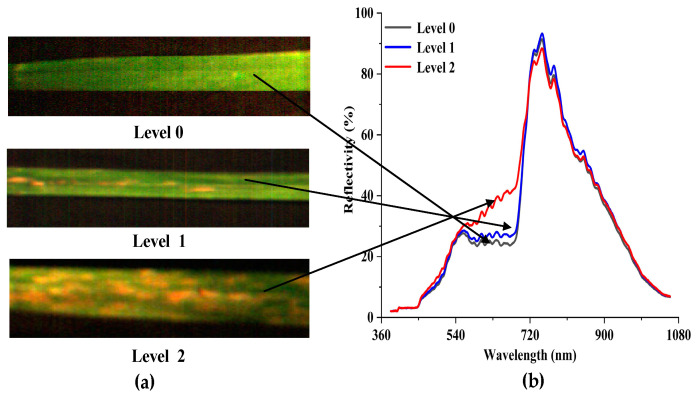
(**a**) False-color composite image showing the three levels and (**b**) Savitzky–Golay (S-G) filter-smoothed data.

The red (680 nm), green (550 nm), and blue (450 nm) bands were composited to show the pseudo-color image in ENVI (Figure 5). The comparison of three disease severities of image (Figure 5a) and spectral (Figure 5b) features can be observed. When checking the image features, it can be found that the leaf of Level 0 has a homogeneous color and texture. For Level 1, the increase of spores affects the textural structure of the original leaf and scattered speckles can be found. The leaf of Level 2 has the highest textural complexity, with more yellow or light-yellow speckles appearing on the leaf. Considering the spectral features, the overall trends of the average spectral reflectivity of the three severities are similar during the specified wavelength bands. Several reflectance peaks for the three levels can be found in the wavelength bands of 555–760 nm. In comparison with Level 2, the spectral curves for Level 0 and Level 1 are extremely similar. Nevertheless, their spectral responses differ in the visible and NIR spectral regions. With the increase of disease severity, the reflectivity also has a large value from 460 to 710 nm. Conversely, the reflectivity shows the opposite trend within the spectral range of 730 to 900 nm.

A hyperspectral spectrometer can collect information based on the measurement of reflected solar energy in hundreds of narrow wavelengths in the human visible spectrum, and also in the NIR and shortwave infrared (SWIR) regions of the solar spectrum. We assume that non-imaging hyperspectral data generally consist of a mixed spectrum in a certain region because of the influences of the background, shadow, soil, etc. Conversely, imaging spectroscopy can simultaneously capture the spectral and image information of materials on the surface. In comparison with high-altitude satellite imagery, ground-based hyperspectral imaging data usually have high spectral and spatial resolutions, which can be very useful in precisely detecting various stresses in green vegetation [24,41]. Additionally, a leaf-scale hyperspectral image is superior in terms of characterizing the PM disease for the canopy-scale datum, due to its simple background. Corresponding band selection and modeling methods can provide a reference for disease detection at the canopy, field, and regional scale. Nevertheless, some issues must be addressed during the processing and application of the hyperspectral dataset, especially the hyperspectral imaging data, such as through feature selection, dimensionality reduction, and sensitivity analysis [29,30,42].

### 3.2. Selection of the Sensitive Spectral Bands

**Figure 6:**
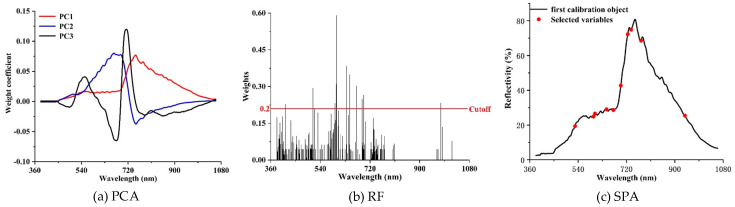
Comparison of characteristic band selection using (**a**) principal component analysis (PCA), (**b**) random forest (RF), and (**c**) the SPA.

Comparisons of selected sensitive spectral bands by the three-dimensional reduction methods are shown in Figure 6 and Table 2. A total of 12 characteristic bands was obtained by the PCA method, and these bands are primarily located around the spectral range between 500 and 850 nm (Figure 6a). A total of 16 characteristic bands were identified through the RF method, among which 11 bands are located between 590 and 700 nm (Figure 6b). In comparison with the above two methods, only 10 bands were selected by the SPA method, and most of them are distributed between 500 and 760 nm (Figure 6c). Considering the selected characteristic bands derived from the three-dimensional reduction methods, it can be found that most of the bands sensitive to wheat PM are located in the visible light range. Nevertheless, there is a significant difference for the band selection of the three methods. For the RF and SPA methods, only one and two bands were located in the NIR spectral range. Conversely, a total of five characteristic bands were selected for the PCA method.

**Figure 7:**
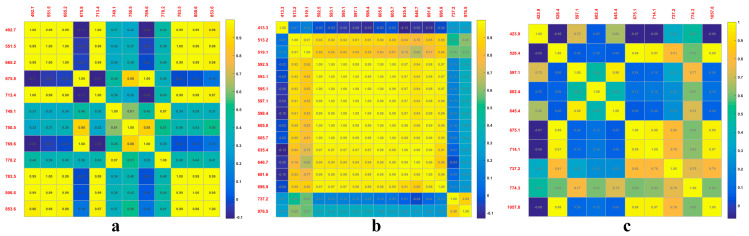
Wavelength correlation for three-dimensional reduction methods: (**a**) PCA; (**b**) RF; (**c**) SPA.

To compare the band selection of PCA, RF, and the SPA, a correlation analysis is shown in Figure 7. It was found that the sensitive bands can be more easily selected by the PCA method (Figure 7a). The characteristic bands selected by the RF method are highly correlated, specifically with 13 wavelengths between 513.2 and 696.9 nm (Figure 7b). The figure shows that the correlation between adjacent bands is higher than that of separable bands, which may be approximately continuous and transferable [43]. The correlation between the characteristic bands obtained by the SPA method is minimal (Figure 7c). The reason for this is that the principle of the SPA is to find the minimum set of redundant information variables from *X* variables, where the selected bands are the group with the lowest correlation between each other [33]. These wavelengths range from 423.9 to 1057.8 nm, with the largest span and the lowest accuracy for the three classifiers, showing that the 10 characteristic bands do not respond strongly to wheat PM.

### 3.3. Models for Identifying Disease Spots on the Leaves

The comparison of classification accuracies is shown in Table 3. The results show that there are no significant differences among the SVM, RF, and PNN models constructed using the original hyperspectral imagery. When comparing the accuracies of the three-dimensional reduction-based models, we can find that the PCA-based SVM model has the highest accuracy of 93.33%. A 5.33% increase is achieved by the dimensionality reduction of hyperspectral bands. The RF model has the same accuracy of 92.00% for the three-dimensional reduction methods, showing that RF has a relatively stable performance. Considering the PNN modeling method, there is no significant accuracy increase for the three-dimensional reduction methods. All of the accuracies are lower than 90.00% and the highest one is just 89.33%.

The running time of the three models was also compared, in addition to the classification accuracies. It was found that the three methods have a significant difference in running time for the same sample data. As can be seen in Table 4, the RF model is the fastest and takes only 0.39 s, while the SVM model requires 9.91 s. Conversely, the PNN model takes the longest time, with the running time of 2327.80 s.

In general, some factors can affect the identification efficiency of wheat PM using hyperspectral imaging, such as the sample quantity, feature selection algorithms, and dimension reduction methods. For example, they usually require more powerful computers and longer running times because of the large data volume and high-dimensional features required for hyperspectral imaging data [44]. Some dimension reduction methods and effective classifiers need to be adopted. In our study, three dimension reduction methods, including PCA, RF, and the SPA, and three classifiers, including the SVM, RF, and the PNN, were selected. It was found that the identification accuracy (Table 3) and running time (Table 4) can be improved by different dimensionality reduction methods, but there are significant differences in the improvement degree.

In terms of the differences of the running time of the three models, the reason for this may be related to the PNN’s four-layer transmission structure [45]. The training and testing of the RF classifier are relatively efficient because the algorithm is a multi-classifier, which can avoid training the classifier multiple times and comprises a small amount of calculation. The applicability and sensitivity must be first evaluated by different dimension reduction methods [46,47,48]. In addition to data processing algorithms and classifiers, the separability of PM leaves with different severities is an important influencing factor. Transitional samples between two levels are easily misclassified. In this study, 45% of the infected area of a leaf was taken as a critical value, and the test could be easily misclassified near the critical value. For example, Levels 0 and 1 are easily misclassified, because it is usually hard to discriminate the differences between a healthy leaf and a slightly infected leaf. The sample quantity is also an affecting factor. When the number of samples per level is small and the number of training samples is small, it is difficult to improve the identification accuracy.

## 4. Conclusions

The PM infection of wheat plants changes the leaf pigment concentration, cell structure, water content, etc., which provides a physical mechanism allowing the detection of such a disease using hyperspectral imaging. Three-dimensional reduction methods, including PCA, RF, and the SPA, and three classifiers, including the SVM, RF, and the PNN, were used to comparatively identify the leaf-scale wheat PM. Consequently, the disease models obtained the highest accuracies for the three classifiers using the PCA dimension reduction method. The SVM model had the highest accuracy, while the RF model obtained stable identification results. Conversely, the PNN model also exhibited a relatively stable performance, but all of the accuracies were lower. In addition to the identification accuracy, we also considered the running time for each model. The PCA-based SVM model had the best performance in terms of detecting leaf-scale wheat PM after comprehensively considering the identification accuracy and running time. This investigation can provide a case study for accurately and finely detecting wheat PM and can also provide a band selection for developing a portable hyperspectral spectrometer.

## Figures and Tables

**Table 1:** Primary steps for performing the successive projections algorithm (SPA)-based dimensionality reduction method.

Operation Procedures for the SPA-Based Dimensionality Reduction Method
(1) Initialization. Perform the first iteration (*n* = 1) and choose any column vector in the spectral matrix. (2) Set *S* is defined as the column vectors that are not included in the band combination, and the projection vector of the selected column vectors on the *S* vector is calculated. (3) Record the serial number of the maximum projection. (4) Take the vector corresponding to the maximum projection ordinal number as the projection vector of the next iteration. (5) *N* = *n* + 1. If *n* < *N*, return to step (2) to continue the projection. (6) The *N* band combinations are obtained according to the different iterations of *N*.

**Table 2:** Selected sensitive bands derived from PCA, RF, and the SPA.

Method	Sensitive Band (nm)
PCA	492.7, 551.5, 665.2, 675.8, 713.4, 749.1, 750.5, 769.6, 778.2, 783.5, 808.6, 853.6
RF	413.3, 513.2, 519.1, 592.5, 593.1, 595.1, 597.1, 598.4, 605, 605.7, 635.4, 646.7, 691.6, 696.9, 737.2, 976.5
SPA	423.9, 528.4, 597.1, 602.4, 645.4, 675.1, 714.1, 737.2, 774.3, 1057.8

**Table 3:** Comparison of identification accuracies for the support vector machine (SVM), RF, and the probabilistic neural network (PNN).

Method	SVM	RF	PNN
None	88.00%	88.00%	86.67%
PCA	93.33%	92.00%	89.33%
RF	88.00%	92.00%	89.33%
SPA	88.00%	92.00%	88.00%

**Table 4:** Comparison of the running time for the SVM, RF, and the PNN.

Method	SVM	RF	PNN
Running time (s)	9.91	0.39	2327.80
